# Pro-Inflammatory Diets Are Associated with Frailty in an Urban Middle-Aged African American and White Cohort

**DOI:** 10.3390/nu15214598

**Published:** 2023-10-29

**Authors:** Marie Fanelli Kuczmarski, May A. Beydoun, Michael F. Georgescu, Nicole Noren Hooten, Nicolle A. Mode, Michele K. Evans, Alan B. Zonderman

**Affiliations:** Laboratory of Epidemiology and Population Sciences, National Institute on Aging, National Institutes of Health, Baltimore, MD 21224, USA; baydounm@mail.nih.gov (M.A.B.); michael.geougescu@nih.gov (M.F.G.); norenhootenn@mail.nih.gov (N.N.H.); nicolle.mode@nih.gov (N.A.M.); evansm@grc.nia.nih.gov (M.K.E.); zondermana@gmail.com (A.B.Z.)

**Keywords:** diet quality, dietary inflammatory index, pre-frail, nutrition, group-based trajectory modeling

## Abstract

Diet quality is a modifiable risk factor for frailty, but research on the association of frailty with dietary inflammatory potential is limited. The objective was to determine associations between diet quality assessed by the dietary inflammatory index (DII) with frailty status over time. Participants with both dietary and frailty data from the longitudinal Healthy Aging in Neighborhoods of Diversity across the Life Span (HANDLS) study were used (*n* = 2901, 43.5% male, 43.8% African American, 48.5 y mean baseline age, with a mean 8.7 y of follow-up). Group-based trajectory modeling identified two frailty (remaining non-frail or being pre-frail/frail over time) and three diet quality trajectory groups (high or medium pro-inflammatory and anti-inflammatory potentials). Multiple logistic regression found both medium pro-inflammatory and anti-inflammatory DII trajectory groups, compared to the high pro-inflammatory group, were positively associated with being non-frail over time for the overall sample, both sexes and races. Kaplan–Meier curves and log-rank test revealed anti-inflammatory DII scores were associated with lower risk for being pre-frail or frail. No longitudinal relationship existed between frailty status at baseline and annualized DII change, a check on reverse causality. This study contributes to our current knowledge providing longitudinal evidence of the link between anti-inflammatory DII score with lower frailty risk.

## 1. Introduction

Frailty is a condition characterized by poor homeostatic responses to stressors yielding functional declines in various physiological systems [1]. It is a strong predictor of falls and fractures in older adults [2,3]. Frailty status fluctuates with time, predicting adverse health outcomes and risk for mortality in individuals with rapidly rising frailty [4,5]. Diet may have a mediating but not a direct effect in the development of frailty [6]. Researchers have suggested that the association of nutrition with frailty may be more robust for dietary quality than adherence to any specific diet [6].

High dietary quality scores seem to have a protective association on the risk for frailty [7,8,9,10]. Consumption of dietary patterns aligned with the Dietary Approaches to Stop Hypertension (DASH) diet, Healthy Eating Index (HEI), and alternate HEI (aHEI) were associated with lower incidence of frailty [11,12,13,14]. Specific dietary components of these healthy diets, such as antioxidants found in fruits and vegetables and dietary fiber provided by whole grains, along with vegetables and fruits, can decrease oxidative stress and inflammation, resulting in a lower risk of frailty [15,16]. In contrast, pro-inflammatory patterns like the Western dietary pattern, a diet characterized by high intakes of processed foods, red meats, refined grains, fat, sucrose, alcohol, and sodium, are associated with higher frailty risk [12].

In addition to diet, other lifestyle behaviors and socio-demographic, clinical and biological factors can contribute to the risk for frailty [17,18]. There are major disparities in frailty prevalence by sex, race, and socioeconomic status. Frailty is more common in women than in men (17.2% vs. 12.9%), in African Americans compared to White people (22.9% vs. 13.8%), and in lower-income compared to higher-income groups (25.8% vs. 5.9%) [19]. There appears to be an increased risk of frailty among adults who experience socioeconomic disadvantage.

In the literature, with the exception of the prospective Healthy Aging in Neighborhoods of Diversity across the Life Span (HANDLS) study, research focused on nutritionally vulnerable diverse urban samples is limited. Among HANDLS study participants, aged 30 to 64 years at baseline, the prevalence of frailty was 7.2% for persons aged 35–44 years, 10.0% for persons aged 45–55 years, and 15.4% in those aged 55–64 years [20]. White adults had a higher prevalence of frailty at younger ages compared to African American adults. Frailty prevalence was higher among those with incomes below poverty status [20]. Importantly, even middle-aged, frail individuals have the lowest survival probability compared to pre-frail and non-frail individuals. Therefore, identifying behavioral and lifestyle factors that contribute to the frailty phenotype may be important avenues for intervention to reduce disparities.

Our previous research has shown the majority of participants in the HANDLS study consume a Western-style dietary pattern. The overall diet quality, assessed by the HEI, of HANDLS study participants was lower than national HEI estimates based on NHANES respondents [21]. Intake of flavonoids, polyphenolic phytochemicals with health-promoting properties, was also lower than that of the United States population [22]. Yet, among the HANDLS study participants, those whose diets had better quality had lower 10-year atherosclerotic cardiovascular health [23] and greater handgrip strength [24]. The role of diet quality in the development of frailty has not yet been explored in this sample.

Although an association between frailty and DII has been reported, this study has the added benefit of employing new statistical methods and of including a more diverse cohort, which is currently lacking in the literature. A group-based trajectory modeling approach, a method using longitudinal data to describe the continuity of different behaviors of groups of persons through time and account for between-individual variation [25], was used. As the HANDLS study has longitudinal data, the application of a life course approach is possible. The objectives of this study are to (1) determine the associations between trajectories of diet quality assessed by the inflammatory potential of diets with trajectories of frailty status and (2) identify diet quality trajectories associated with high probability of remaining non-frail over time. We hypothesize that an anti-inflammatory diet would be associated with lower risk for frailty.

## 2. Methods

### 2.1. HANDLS Study

A description of the population-based cohort study, named HANDLS, has been published and can be found on the HANDLS website [26]. The study aims were to determine the role of race and socioeconomic status in health disparities. The sample of African American and White men and women was recruited as an area probability sample. Participants resided in 13 pre-determined Baltimore, Maryland neighborhoods in the USA. Initiated in August 2004, the baseline wave ended in March 2009. The cohort included 3720 urban-dwelling individuals aged 30–64 years at baseline.

### 2.2. Study Participants

The sample in this study was obtained from the adults interviewed and examined in Waves 1, 3, and 4 of the HANDLS study. Waves 3 and 4, the first and second in-person follow-up waves, were conducted between June 2009 and July 2013 and between September 2013 and September 2017, respectively. In this article, Wave 1 is referred to as Visit (*v*) 1, Wave 3 as *v* 2, and Wave 4 as *v* 3. To be included in this study, participants needed to have complete data at either visit on both diet quality indices and frailty scores, 2901 of the initial 3720 participants recruited at *v* 1 met these criteria. Written informed consent was obtained from each participant. Participants were compensated monetarily for their participation. The Human Institutional Review Board at the National Institutes of Health approved the study protocol.

### 2.3. Participant Characteristics

Participants self-reported their race as African American or White; sex assigned at birth was coded as male or female. Participants were categorized as above or below poverty status defined by 125% of the 2004 United States (US) Health and Human Services Poverty Guidelines at baseline enrollment [27]. Education was coded as less than high school, high school or GED, or more than high school. Cigarette smokers and users of marijuana, opiate, and/or cocaine drugs were coded as current or never/former users. Allostatic load estimation for the HANDLS study sample has been described in detail elsewhere [28]. Allostatic load combines several risk indicators, namely cardiovascular (systolic and diastolic blood pressure, pulse rate), metabolic (total cholesterol, HDL cholesterol, glycated hemoglobin (HbA1c), sex-specific waist-to-hip ratio), and inflammatory (serum ALB and high-sensitivity C-reactive protein). Allostatic load was included since there is life-course evidence that allostatic load in midlife is associated with frailty in later years [29,30].

### 2.4. Dietary Collection Method

The US Department of Agriculture (USDA) Automated Multiple-Pass Method (AMPM) was used to collect two 24 h recalls at each visit, scheduled 4–10 days apart across all days of the week [31]. All recalled foods and beverages were assigned USDA food codes from the USDA Food and Nutrient Database for Dietary Studies (FNDDS). Foods and beverages reported in *v* 1 were coded using FNDDS 3.0 (2005–2006), for *v* 2, FNDDS 5.0 (2009–2010) and for *v* 3, FNDDS 2013–2014 [32]. Both 24 h recalls in HANDLS *v* 1 were collected in person. For *v* 2 and *v* 3, in-person interviews were conducted for the first 24 h recall and telephone interviews were conducted to obtain the second 24 h recall. To assist the study participants with estimation of the portions of foods consumed, an illustrated food model booklet, as well as other aids, namely a ruler, measuring cups, and spoons, were available during all dietary interviews. Only those participants with two 24 h dietary recalls were included in this study. Of the 3720 baseline participants, two 24 h recalls were collected from 2177 adults at *v* 1, 2140 at *v* 2, and 2066 at *v* 3.

### 2.5. Dietary Inflammatory Index (DII)

The inflammatory potential of the diet was calculated using the following 35 of the original 45 parameters [33]: energy, alcohol, protein, carbohydrate, dietary fiber, total fat, saturated fat, monounsaturated fat, polyunsaturated fat, omega-3 fatty acids, omega-6 fatty acids, cholesterol, 11 vitamins (A, B_6,_ B_12_, β-carotene, C, D, E, folic acid, niacin, riboflavin, thiamin), 4 minerals (iron, magnesium, selenium, zinc), 6 flavonoid classes (flavan-3-ol, flavones, flavonols, flavanones, anthocyanidins, isoflavones), caffeine, and tea. The excluded parameters were trans fat and 9 spices. Trans fat was not included in the USDA FNDDS and no information was gathered on spices during the 24 h recalls. The possible maximal anti-inflammatory DII score was −10.44 for the HANDLS study participants, and the maximal pro-inflammatory DII score was +10.44 when applying the global composite database to our data. Typically, the lower the DII score, the more anti-inflammatory the dietary pattern.

### 2.6. Frailty

Frailty was determined using a modified FRAIL scale, which is based on 5 domains, namely fatigue, resistance, ambulation, number of illnesses and loss of weight [20,34]. Fatigue was measured from responses to “I could not get going” (item 20) of the Center for Epidemiologic Studies Depression scale (CES-D) [35]. Participant reports of any difficulty walking up 10 stairs and difficulty walking a quarter of a mile were used to assess resistance and ambulation, respectively. Illness was assessed as positive reports of 5 or more conditions out of 11 conditions included in the structured medical history. The conditions were hypertension, diabetes, cancer, chronic lung disease, heart attack, congestive heart failure, angina, asthma, arthritis, stroke, and kidney disease. Loss of weight was assessed from participant response to “I did not feel like eating or my appetite was poor” (item 2) of the CES-D. Weight loss was considered present when participants responded occasionally (3 ± 4 days a week) or mostly (5 ± 7 days a week). The calculation of the scores was based on the components present, described in detail elsewhere [20]. Scores could range from 0 to 5, where 0 meant that all components were absent and 5 meant that all components were present, and participants were categorized into three groups—non-frail (score = 0), pre-frail (score = 1 or 2), and frail (score = 3, 4 or 5). There were 3050 of the original 3720 *v* 1 participants who had frailty scores on at least one of 3 visits, specifically 2815 for *v* 1, 1868 for *v* 2 and 2091 for *v* 3.

### 2.7. Statistical Analyses

Study sample characteristics were compared across sex and across race. Multiple imputations (5 imputations, 10 iterations) were conducted using chained equations for the non-exposure and non-outcome variables with missing data, namely education and allostatic load. To this end, bivariate multinomial logit models with imputed data for categorical variables and bivariate linear regression models for continuous variables were used. Sex and race were the only predictors in these models.

Using available data from the 3 visits, the *traj* and *trajplot* Stata plugin for estimating group-based trajectory modeling (GBTM) were used to create the DII trajectory groups [36,37], as well as the frailty score trajectory groups (0 = non-frail; 1 = pre-frail; 2 = frail). The plugin is adapted from a well-established SAS procedure [36]. It identifies groups of persons with similar developmental trajectories over time. This group-based approach utilizes a multinomial modeling strategy and maximum likelihood to estimate model parameters, with maximization achieved by the quasi-Newton procedure. We specified a censored normal distribution for the DII and a zero-inflated Poisson regression for the frailty score, with intercept (0), linear (1), quadratic (2), and cubic (3) orders for each group trajectory. Group-based trajectories over time were displayed with 95% confidence intervals (CI). We defined up to three groups for DII, and three groups were also attempted for frailty scores.

Multiple logistic regression was performed with the outcome “remaining non-frail” vs. “all others” based on the GBTM grouping. The DII GBTM trajectory groups were reordered such that 1 = pro-inflammatory, 2 = medium, and 3 = anti-inflammatory. The modeling strategy first adjusted for age at *v* 1, sex, race and poverty status, and second further adjusted for education, smoking, drug use, and allostatic load.

Using another modeling strategy, time to frailty (frail or pre-frail vs. non-frail) was considered as an outcome of interest and predicted by DII trajectory groups using Cox proportional hazard regression, adjusting for a similar set of covariates, applied to the data in a person–period format. This format allowed for the exclusion of individuals who were pre-frail or frail at *v* 1 from the risk set or who were pre-frail or frail at *v* 2 from the risk set for the follow-up to *v* 3.

Kaplan–Meier survival curves are presented to compare frailty- or pre-frailty free probability across DII trajectory groups (low-, medium- and high-quality diets), along with a log-rank test for equality of survivor function (Chi-square test, 2 degrees of freedom) with associated *p*-value.

Finally, mixed-effects linear regression models were run using the DII scores as outcomes and frailty status as the predictor to examine the effect of frailty status at *v* 1 on annualized rate of change in time-dependent DII. The number of years elapsed from *v* 1 through *v* 3 was used as the TIME variable in model. This TIME parameter was interacted with the frailty at the baseline group as were all potentially confounding covariates. The main effects of the predictor were interpreted as the effect of predictors on the DII outcome at time zero (or *v* 1). Random effects were added to both TIME and the intercept to allow for individual-level variability in baseline and annualized change in DII. The model assumed missingness in outcome at random.

## 3. Results

### 3.1. Sample Characteristics

The mean age of the sample at *v* 1 was 48.5 years with no significant differences in age by race and sex (Table 1). The mean age at *v* 2 was 53.0 ± 0.2 years and at *v* 3 was 56.6 ± 0.2 years. The mean number of years between *v* 1 and *v* 2 was 4.66 ± 0.02. The mean number of years between *v* 1 and *v* 3 was 8.67 ± 0.04.

Females and African American adults had greater likelihood of having incomes below poverty compared to males and White adults (*p* < 0.001), respectively. Incomes below poverty were reported by approximately 41% of the sample, ranging from 32 to 44% across sex and race groups (Table 1). More males, compared to females, were current smokers (55% vs. 44%, *p* < 0.001) and users of marijuana, cocaine and/or heroin drugs (24% vs. 14%, *p* < 0.001). More African American adults in comparison to White participants were smokers (51% vs. 46%, *p* < 0.001) and current drug users (22% vs. 13%, *p* < 0.001). Although there were no differences in education by sex, there were racial differences. A smaller percentage of African American adults had less than high school education as well as more than high school attainment compared to the White adult participants (Table 1). Mean (±SE) allostatic load of the overall sample was 1.94 (±0.03) with no differences by sex or by race (Table 1).

### 3.2. Frailty Categories and Group Trajectories

The percentage of the sample in the frail group increased over time (Table 2). At each visit, there were more women in the pre-frail and frail categories than men. However, there were no racial differences in the percentage of persons in the pre-frail or frail groups (Table 2).

**Figure 1:**
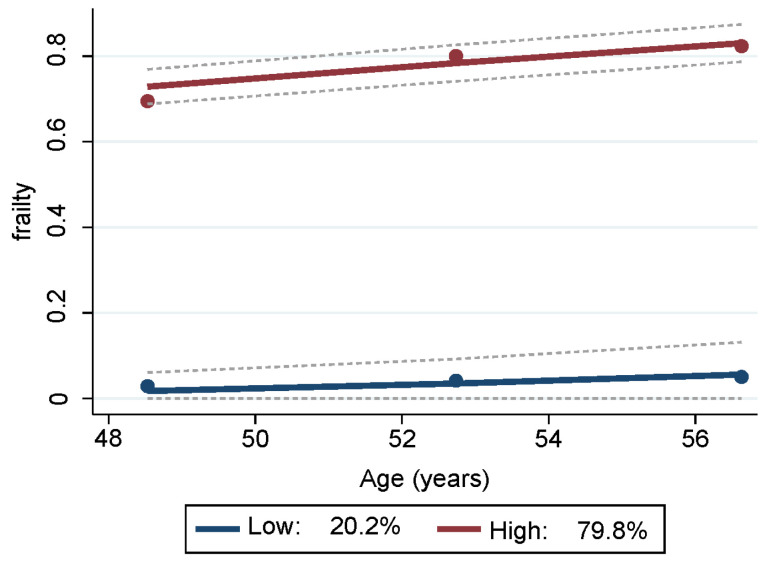
Trajectories of frailty status of HANDLS study sample over time. Notes: Y axis represents average proportion frail at each mean age per wave. Blue represents mostly “non-frail throughout follow-up” and red represents “pre-frail/frail throughout follow-up”. Dashed lines are 95% pointwise confidence intervals on the estimated trajectories. *Note:* HANDLS, Healthy Aging in Neighborhoods of Diversity across the Life Span.

Two trajectory groups for frailty were identified from the GBTM analyses (Table S1) and depicted in Figure 1. One group trajectory comprised mostly non-frail HANDLS study participants, representing 20.16% (SE = 2.12) of the sample. The other group trajectory comprised the majority of the sample, namely 79.84% (SE = 2.12), and consisted of participants who were either pre-frail or frail. Depicted in blue in Figure 1, the lower group trajectory are those individuals who were mostly non-frail throughout all visits. Depicted in red, the upper group trajectory consisted of persons who were classified as pre-frail and/or frail at the three different visits.

### 3.3. Dietary Quality Group Trajectories

**Figure 2:**
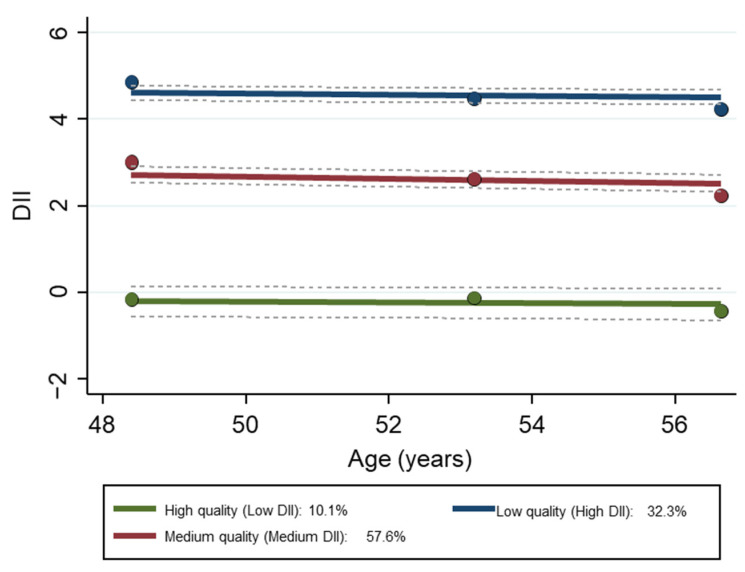
*Group trajectories of DII of the HANDLS study sample over time, N = 2901.* Dashed lines are 95% pointwise confidence intervals on the estimated trajectories. *Note*: the Y axis is the original DII score. Abbreviations: DII, Dietary Inflammatory Index; HANDLS, Healthy Aging in Neighborhoods of Diversity across the Life Span.

Three diet quality trajectory groups were identified (Table S2). They were labeled by their inflammatory potential—high, medium, or low quality, according to their DII scores. Low quality reflects anti-inflammatory (negative) scores while medium and low quality reflect pro-inflammatory (positive) scores. The mean (±SE) DII scores by trajectory for each visit are provided in Table S3 and displayed in Figure 2.

The empirical Bayes estimates of the DII and slope across visits were based on all available data on dietary intakes from 2–24 h recalls. The mean (±SE) empirical Bayes estimator for DII for the overall sample was 3.30 ± 0.02 (95% CI [3.26, 3.34]), with a slope of −0.0801 (±0.0002). For women, the mean (±SE) empirical Bayes estimator for DII was 3.46 ± 0.03 (95% CI [3.41, 3.52]), with a slope of −0.0792 (±0.0002); for men, it was 3.08 ± 0.03 (95% CI [3.02, 3.14]), with a slope of −0.0812 (±0.0002). For African American adults, the mean (±SE) empirical Bayes estimator for DII was 3.44 ± 0.02 (95% CI [3.39, 3.48]), with a slope of −0.0794 (±0.0002); for White participants, it was 3.09 ± 0.04 (95% CI [3.02, 3.17]) with a slope of −0.0810 (±0.0003). The negative slope indicates that the DII score was moving in the direction towards anti-inflammatory, suggesting improvement in diet quality with time.

### 3.4. Findings Based on Multiple Logistic Regression with Remaining Non-Frail as Outcome

The results of Model 1 and Model 2 of the multiple logistic regression using the overall sample found that both medium pro-inflammatory and anti-inflammatory DII group trajectories (red and green trajectories in Figure 2) were positively and significantly associated with the remaining non-frail free trajectory compared to the high pro-inflammatory group (blue trajectory in Figure 2) (Table 3). Model 1 adjusted for age, sex, race, and poverty status, while Model 2 further adjusted Model 1 for education, smoking, drug use, and allostatic load.

Stratified logistic regression analyses performed separately by sex and by race found the same significant associations between frailty status and diet quality as the overall sample (Tables S4 and S5). No significant interaction of either sex or race with DII trajectory groups and frailty status were found.

### 3.5. Findings Based on Proportional Hazards Regression with Time to Frailty as Outcome

The results of the Cox proportional hazards regression models are provided in Table 4. The hazard ratio (HR) for the high diet quality trajectory (anti-inflammatory DII) group was 0.35 and the HR for the medium diet quality (medium pro- inflammatory DII) trajectory group was 0.81 compared to low diet quality (high pro-inflammatory DII) trajectory. These models controlled for age, sex, race, poverty status, education, smoking, drug use and allostatic load.

**Figure 3:**
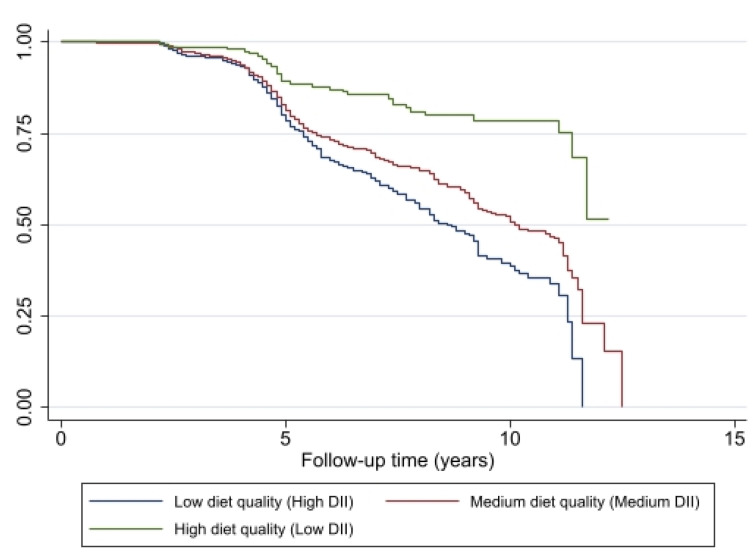
Association of change in diet quality, measured by DII, with remaining non-frail (y axis) for HANDLS study participants (*N* = 1349, *N*′ = 2065, *n* = 549). Note: *N* = Number of subjects included in the analysis; *N*′ = Number of observations included in the analysis, *n* = total number of incident frail/pre-frail.

The probability for remaining non-frail was lower at each year of follow-up among individuals with pro-inflammatory diets (high and medium DII) compared with individuals with an anti-inflammatory diet (low DII), based on the Kaplan–Meier curves and the log-rank test (*p* < 0.001) (Figure 3). The lowest probability for being pre-frail or frail was associated with better diet quality, a diet with a DII score indicating an anti-inflammatory potential.

### 3.6. Findings Based on Mixed-Effects Regression with DII Scores as Outcome

The results of the mixed-effect regression analyses revealed that frailty status at *v* 1 had only a cross-sectional association with DII at *v* 1, and had no detectable relationship with annualized change in DII between *v* 1 and *v* 3 (Table S6). The first analysis, Model 1, adjusted for race, sex, age, and poverty status. The second analysis, Model 2, further adjusted Model 1 for education, smoking, drug use, and allostatic load. Similar to the findings for the overall sample, the same findings were observed for both models when performed by sex.

## 4. Discussion

The study findings provide evidence that groups of individuals belonging to the trajectories of pro-inflammatory diets, as indicated by high DII scores, were more likely to be in the pre-frail or frail trajectories after adjusting for health-related and sociodemographic covariates. This association between the inflammatory potential of the diet and frailty was observed for both sexes and both races. Using the GBTM approach was simpler than more complex models, yielding results that were consistent with publications using more traditional approaches. Shivappa and colleagues found that in U.S. men, but not women, individuals in the most pro-inflammatory category (highest DII quartile scores) had a 37% higher risk of developing frailty during an eight-year follow-up period [38]. Using data from the Framingham Heart Study, Millar and colleagues found a pro-inflammatory diet was associated with increased odds of frailty in middle-aged and older adults over an approximate 12-year follow-up [39]. Our study results are also consistent with the findings of Resciniti and colleagues who reported that US adults examined in the NHANES, 2007–2014, categorized in the highest quintile of DII scores, compared to the lowest quintile of DII scores, were more likely to be pre-frail or frail [40]. Among Spanish adults 60 years and older enrolled in the Seniors-ENRICA cohort, DII predicted frailty with those in the highest tertile having higher risk of frailty compared to individuals in the lowest DII tertile [41].

This study expands upon previous knowledge of the association of diet quality with frailty, providing evidence of diet-related disparities resulting in differences in burden in terms of frailty among the nutritionally vulnerable African American and White persons enrolled in the HANDLS study. Diet intakes reported in the HANDLS study with more anti-inflammatory potential, even though markedly lower than the possible maximal DII score based on the global composite database, were associated with a reduced risk for frailty in this study. The findings of the Cox proportional hazard regression suggest a diet with anti-inflammatory potential provides a 65% reduction in risk for developing frailty compared to consuming a diet with high pro-inflammatory potential. A diet of medium quality, defined in this study as medium pro-inflammatory DII, provides a 20% reduction in risk compared to the diet with high pro-inflammatory DII scores. Diet-related disparities contribute to differences in health and disease. Contributors to diet-related disparities include but are not limited to socioeconomic status, race, environment, and cultural preferences [42].

Diet quality evaluated by criterion different from inflammatory potential, such as adherence to national dietary guidelines like the Healthy Eating Index, or reduction in risk for chronic disease such as the Mediterranean Diet score and DASH (Dietary Approaches to Stop Hypertension) scores have found similar associations with frailty [7,10,11,12,43]. These relationships most likely reflect the inclusion of antioxidants, polyphenols, dietary fiber and other nutrients and dietary components provided by high-quality diets [8,14]. Like the DII, the pathophysiological links of these various diet quality measures support the pathogenic implication of inflammatory mechanisms in frailty.

Frailty is a dynamic condition, resulting in the movement between frailty states by some participants over follow-up visits. In this study, GBTM was used to determine group means at each visit. However, latent trajectory modeling could be used to study transitions in and out of frailty states. Furthermore, the potential for reverse causation could be possible, as dietary consumption and nutritional status may be altered if persons are pre-frail or frail. Supplementary analyses found that frailty status at the first visit was not associated with the annualized rate of change in DII scores, suggesting frailty state was not a predictor of change in diet quality.

Some of the strengths of this study include the fact that it was a longitudinal prospective cohort study of a reasonably large sample, which included both biological sexes and two races. Few dietary studies include socio-economically diverse African American and White adults. We also adjusted for a broad range of lifestyle, socioeconomic, and clinical factors. This study had dietary and frailty assessments at multiple time points, which provided the additional benefit of assessing the associations over time. Another strength was the use of a validated dietary collection method, namely the AMPM.

Despite its strengths, there are limitations to our study. The DII only captured 35 of the 45 food parameters in the original calculation by Shivappa et al. [33]. In our opinion, DII predictive capability was not decreased given we had more than 28 parameters, the minimum number suggested by Shivappa et al. [33]. Notably, the majority of omitted food parameters in our study’s DII calculation, namely the spices, are not commonly consumed in an American diet. The inclusion of spices in the DII score would require not only the addition of spices to nutrient databases but also the knowledge of all recipes from both manufacturers and individuals preparing foods, as well as spice use at the table. It seems calculations of estimated spice intakes may present a challenge. The exclusion of trans fat from our DII score, an omission noted by other researchers [44], reflects a lack of this fat in nutrient databases. Furthermore, in the USA, partially hydrogenated oils from processed foods, the source of artificial trans fat, were to be removed from the food supply [45]. Therefore, it appears that this DII component may no longer be relevant. Additionally, the dietary DII scores were based on self-reported dietary data which can be affected by the misreporting of intake portions and subject to social desirability and do not include nutritional supplement intakes. Adjustments for physical activity could not be made since the inclusion of activity assessments began at *v* 2. Lastly, GBTM involves some subjective decisions regarding the number of groups, the decisions whether to have quadratic terms for some or all of them, and the determination of the best-fit combination.

## 5. Conclusions

In conclusion, this study contributes to our current knowledge regarding the relationship of dietary inflammation to frailty. The consumption of a diet with more inflammatory potential was associated with being pre-frail or frail in this cohort of African American and White male and female adults over a mean 8.7-year follow-up. Even though the anti-inflammation potential of their dietary patterns scored much lower than the possible maximum, our findings were consistent with those of other researchers [16,39,40,41]. A diet rich in dietary antioxidants, fiber, vegetables, and fruits and low in ultra-processed foods appears to play a significant role in the prevention of frailty.

## Figures and Tables

**Table 1:** Sample characteristics at Visit 1 of HANDLS study, overall, by sex and by race.

Characteristic	Overall	Sex			Race		
		Males	Females	*p* ^a^	African American	White	*p*
	*N* = 2901	*N* = 1261	*N* = 1640		*N* = 1724	*N* = 1177	
Age, *v* 1, X ± SE	48.5 ± 0.2	48.4 ± 0.3	48.5 ± 0.2	0.573	48.3 ± 0.2	48.7 ± 0.3	0.312
Sex, % Males	43.5	-	-		43.3	43.8	0.796
Race, % African American	59.4	59.2	59.6	0.796	-	-	
% below poverty status ^b^	41.2	37.6	44.0	0.001	47.7	31.7	<0.001
Education, <HS, %	6.5	7.3	5.9	0.248	4.8	9.0	<0.001
Education, HS, %	60.2	60.9	59.6	reference	64.4	53.9	reference
Education, >HS, %	33.3	31.8	34.5	0.214	30.8	37.1	<0.001
Current smokers, %	48.8	54.9	44.1	<0.001	51.0	45.5	0.010
Current drug users, %	18.2	23.9	13.9	<0.001	21.8	13.1	<0.001
Allostatic load, X ± SE	1.94 ± 0.03	1.91 ± 0.05	1.96 ± 0.04	0.466	1.90 ± 0.04	1.99 ± 0.04	0.146

Abbreviation: HANDLS, Healthy Aging in Neighborhoods of Diversity Across the Life Span; HS, High School (includes earned high school equivalency); SE, Standard Error; *v* 1, *visit* 1. ^a^ *p*-values based on bivariate multinomial logit models with imputed data for categorical variables and bivariate linear regression models for continuous variables. Sex and race were the only predictors in these models, entered alternatively. ^b^ Defined as < 125% 2004 HHS Federal poverty guidelines.

**Table 2:** Percentage (±SE) of HANDLS study participants by frailty category for the overall sample and by sex and race at each visit.

Frailty	Overall	Sex			Race		
		Males	Females	β ± SE	African Americans	White	β ± SE
	*N* = 2901	*N* = 1261	*N* = 1640		*N* = 1724	*N* = 1177	
***Visit 1***							
Non-frail	54.1 ± 1.0	62.1 ± 1.4	48.0 ± 1.3	Referent group	54.2 ± 1.3	53.9 ± 1.5	Referent group
Pre-frail	35.9 ± 0.9	31.1 ± 1.4	39.6 ± 1.3	−0.50 ± 0.08 ***	36.5 ± 1.2	35.1 ± 1.4	0.03 ± 0.08
Frail	10.0 ± 0.6	6.7 ± 0.7	12.4 ± 0.8	−0.87 ± 0.14 ***	9.2 ± 0.7	11.0 ± 0.9	−0.18 ± 0.13
***Visit 2***							
Non-frail	49.1 ± 1.2	56.4 ± 1.8	43.8 ± 1.5	Referent group	49.6 ± 1.5	48.4 ± 1.8	Referent group
Pre-frail	38.2 ± 1.1	36.3 ± 1.7	39.6 ± 1.5	−0.34 ± 0.10 **	38.3 ± 1.5	38.1 ± 1.7	−0.02 ± 0.10
Frail	12.7 ± 0.8	7.3 ± 0.9	16.6 ± 1.1	−1.07 ± 0.17 ***	12.1 ± 1.0	13.5 ± 1.2	−0.14 ± 0.15
***Visit 3***							
Non-frail	48.6 ± 1.1	55.8 ± 1.7	43.6 ± 1.4	Referent group	49.7 ± 1.4	47.0 ± 1.7	Referent group
Pre-frail/Frail	39.7 ± 1.1	33.0 ± 1.6	39.2 ± 1.4	−0.42 ± 0.10 ***	36.5 ± 1.4	36.8 ± 1.7	−0.06 ± 0.10
Frail	14.7 ± 0.8	11.2 ± 1.1	17.2 ± 1.1	−67.4 ± 0.14 ***	13.8 ± 1.0	16.2 ± 1.3	−0.21 ± 0.13

Abbreviation: β is the Log_e_(OR) from a multinomial logistic regression model with frailty status as the outcome and sex or race as the only predictor. HANDLS, Healthy Aging in Neighborhoods of Diversity across the Life Span; SE, Standard Error, ** *p* = 0.001, *** *p* < 0.001.

**Table 3:** Association between DII and frailty GBTM trajectories: Multiple logistic regression model, HANDLS study.

*N* = 2901	Loge (OR)	SE	*p*
**Model 1**			
Pre-frail or frail	Base outcome ^a^		
***Remaining non-frail trajectory = main outcome***			
*DII trajectory*	Referent: Group 1		
Medium vs. High DII	0.662	0.116	<0.001
Low vs. High DII	1.569	0.171	<0.001
Age *v* 1	0.009	0.005	0.076
Sex, Male	0.130	0.095	0.171
Race, African American	0.282	0.099	0.004
Poverty, <125% poverty	−0.886	0.106	<0.001
**Model 2**			
Pre-frail or frail	Base outcome		
***Remaining non-frail trajectory = main outcome***			
*DII trajectory*	Referent: Group 1		
Medium vs. High DII	0.570	0.120	<0.001
Low vs. High DII	1.291	0.181	<0.001
Age *v* 1	0.010	0.006	0.062
Sex, Male	0.265	0.100	0.008
Race, African American	0.317	0.103	0.002
Poverty, <125% poverty	−0.741	0.111	<0.001
*Education*	Referent: <High School		
High School	0.267	0.252	0.289
>High School	0.405	0.256	0.118
Current smoker *v* 1	−0.561	0.128	<0.001
Drug User *v* 1	−0.429	0.161	0.010
Allostatic load	−0.209	0.050	<0.001

Abbreviations: SE are the standard errors for the coefficient. DII, Dietary Inflammatory Index; GBTM, Group-Based Trajectory Modeling; HANDLS, Healthy Aging in Neighborhoods of Diversity Across the Life Span; *v* 1, visit 1. ^a^ The base outcome group is used as the reference group and the coefficients for all other outcome groups describe how the independent variables are related to the probability of being in that outcome group versus the reference group.

**Table 4:** Association of DII GBTM trajectory groups with incidence of frailty or pre-frailty. Cox proportional hazards model, HANDLS study.

*N* = 2065	Coefficient	SE	*p*	HR	LCL	UCL
*DII trajectory*						
Medium vs. High DII	−0.215	0.098	0.028	0.806541	0.665591	0.977341
Low vs. High DII	−1.042	0.207	<0.001	0.352748	0.235106	0.529258
Age *v* 1	0.014	0.005	0.004	1.014098	1.004209	1.024085
Sex	−0.286	0.089	0.013	0.751263	0.631006	0.894438
Race	−0.286	0.091	0.002	0.751263	0.628537	0.897951
Poverty status	0.139	0.094	0.141	1.149124	0.955768	1.381597
*Education*						
High School	0.050	0.197	0.800	1.051271	0.714537	1.546694
>High School	−0.064	0.209	0.760	0.938005	0.622731	1.412894
Current smoker *v* 1	0.329	0.100	0.001	1.389578	1.14225	1.690459
Drug User *v* 1	0.222	0.116	0.057	1.248571	0.994654	1.567309
Allostatic load	0.102	0.040	0.014	1.107383	1.023881	1.197696

*Notes:* DII, Dietary inflammatory index; HANDLS, Healthy Aging in Neighborhoods of Diversity Across the Life Span; HR, Hazard Ratio; LCL, Lower Confidence Limit of the 95% CI of HR; SE, Standard error; UCL, Upper Confidence Limit of the 95% CI of HR; *v* 1, *visit* 1.

## Data Availability

Data are available upon request to researchers with valid proposals who agree to the confidentiality agreement as required by our Institutional Review Board. We publicize our policies on our website https://handls.nih.gov (accessed on 28 October 2023). Requests for data access may be sent to Alan Zonderman (co-author) or the study manager, Jennifer Norbeck, at norbeckje@mail.nih.gov.

## Supplementary Materials

The following supporting information can be downloaded at: https://www.mdpi.com/article/10.3390/nu15214598/s1, Table S1. Group-based Trajectory Modeling output for frailty; Table S2. Group-based Trajectory Modeling output for Dietary Inflammatory Index; Table S3. Percentage of sample by group trajectory and mean (±SE) DII scores by group trajectory for each HANDLS study visit (*n* = 2901); Table S4. Association of DII GBTM trajectories with frailty GBTM trajectories, by sex, Multiple Logistic Regression models, HANDLS study (*N* = 1349, *N*’ = 2065, *n* = 549); Table S5. Multiple Logistic Regressions of the association of diet quality and frailty by race; Table S6. Mixed-effect regression of the association of *v* 1 frailty status and DII continuous score, HANDLS study.

## References

[1] Gimeno-Mallench L., Sanchez-Morate E., Parejo-Pedrajas S., Mas-Bargues C., Inglés M., Sanz-Ros J., Román-Domínguez A., Olaso G., Stromsnes K., Gambini J. (2020). The Relationship between Diet and Frailty in Aging. Endocr. Metab. Immune Disord. Drug Targets.

[2] Jang I.-Y., Jung H.-W., Lee H.Y., Park H., Lee E., Kim D.H. (2020). Evaluation of Clinically Meaningful Changes in Measures of Frailty. J. Gerontol. Ser. A Biol. Sci. Med. Sci..

[3] Howlett S.E., Rutenberg A.D., Rockwood K. (2021). The degree of frailty as a translational measure of health in aging. Nat. Aging.

[4] Stow D., Matthews F.E., Hanratty B. (2018). Frailty trajectories to identify end of life: A longitudinal population-based study. BMC Med..

[5] Thompson M.Q., Theou O., Tucker G.R., Adams R.J., Visvanathan R. (2019). Recurrent Measurement of Frailty Is Important for Mortality Prediction: Findings from the North West Adelaide Health Study. J. Am. Geriatr Soc..

[6] Ni Lochlainn M., Cox N.J., Wilson T., Hayhoe R.P.G., Ramsay S.E., Granic A., Isanejad M., Roberts H.C., Wilson D., Welch C. (2021). Nutrition and Frailty: Opportunities for Prevention and Treatment. Nutrients.

[7] Capurso C., Bellanti F., Lo Buglio A., Vendemiale G. (2019). The Mediterranean Diet Slows Down the Progression of Aging and Helps to Prevent the Onset of Frailty: A Narrative Review. Nutrients.

[8] Feart C. (2019). Nutrition and frailty: Current knowledge. Prog. Neuro-Psychopharmacol. Biol. Psychiatry.

[9] Parsons T.J., Papachristou E., Atkins J.L., Papacosta O., Ash S., Lennon L.T., Whincup P.H., Ramsay S.E., Wannamethee S.G. (2019). Physical frailty in older men: Prospective associations with diet quality and patterns. Age Ageing.

[10] Struijk E.A., Hagan K.A., Fung T.T., Hu F.B., Rodríguez-Artalejo F., Lopez-Garcia E. (2020). Diet quality and risk of frailty among older women in the Nurses’ Health Study. Am. J. Clin. Nutr..

[11] Ward R.E., Orkaby A.R., Chen J., Hshieh T.T., Driver J.A., Gaziano J.M., Djousse L. (2020). Association between Diet Quality and Frailty Prevalence in the Physicians’ Health Study. J. Am. Geriatr. Soc..

[12] Jayanama K., Theou O., Godin J., Cahill L., Shivappa N., Hébert J.R., Wirth M.D., Park Y.-M., Fung T.T., Rockwood K. (2021). Relationship between diet quality scores and the risk of frailty and mortality in adults across a wide age spectrum. BMC Med..

[13] Hengeveld L.M., Wijnhoven H.A.H., Olthof M.R., Brouwer I.A., Simonsick E.M., Kritchevsky S.B., Houston D.K., Newman A.B., Visser M. (2019). Prospective Associations of Diet Quality with Incident Frailty in Older Adults: The Health, Aging, and Body Composition Study. J. Am. Geriatr. Soc..

[14] Watanabe D., Kurotani K., Yoshida T., Nanri H., Watanabe Y., Date H., Itoi A., Goto C., Ishikawa-Takata K., Kimura M. (2022). Diet quality and physical or comprehensive frailty among older adults. Eur. J. Nutr..

[15] Rashidi Pour Fard N., Amirabdollahian F., Haghighatdoost F. (2019). Dietary patterns and frailty: A systematic review and meta-analysis. Nutr. Rev..

[16] Fung T.T., Struijk E.A., Rodriguez-Artalejo F., Willett W.C., Lopez-Garcia E. (2020). Fruit and vegetable intake and risk of frailty in women 60 years old or older. Am. J. Clin. Nutr..

[17] Feng Z., Lugtenberg M., Franse C., Fang X., Hu S., Jin C., Raat H. (2017). Risk factors and protective factors associated with incident or increase of frailty among community-dwelling older adults: A systematic review of longitudinal studies. PLoS ONE.

[18] Hoogendijk E.O., Afilalo J., Ensrud K.E., Kowal P., Onder G., Fried L.P. (2019). Frailty: Implications for clinical practice and public health. Lancet.

[19] Bandeen-Roche K., Seplaki C.L., Huang J., Buta B., Kalyani R.R., Varadhan R., Xue Q.-L., Walston J.D., Kasper J.D. (2015). Frailty in Older Adults: A Nationally Representative Profile in the United States. J. Gerontol. Ser. A.

[20] Griffin F.R., Mode N.A., Ejiogu N., Zonderman A.B., Evans M.K. (2018). Frailty in a racially and socioeconomically diverse sample of middle-aged Americans in Baltimore. PLoS ONE.

[21] Fanelli Kuczmarski M., Cotugna N., Pohlig R.T., Beydoun M.A., Adams E.L., Evans M.K., Zonderman A.B. (2017). Snacking and Diet Quality Are Associated With the Coping Strategies Used By a Socioeconomically Diverse Urban Cohort of African-American and White Adults. J. Acad. Nutr. Diet.

[22] Fanelli Kuczmarski M., Sebastian R.S., Goldman J.D., Murayi T., Steinfeldt L.C., Eosso J.R., Moshfegh A.J., Zonderman A.B., Evans M.K. (2018). Dietary Flavonoid Intakes Are Associated with Race but Not Income in an Urban Population. Nutrients.

[23] Fanelli Kuczmarski M., Bodt B.A., Stave Shupe E., Zonderman A.B., Evans M.K. (2018). Dietary Patterns Associated with Lower 10-Year Atherosclerotic Cardiovascular Disease Risk among Urban African-American and White Adults Consuming Western Diets. Nutrients.

[24] Fanelli Kuczmarski M., Pohlig R.T., Stave Shupe E., Zonderman A.B., Evans M.K. (2018). Dietary Protein Intake and Overall Diet Quality Are Associated with Handgrip Strength in African American and White Adults. J. Nutr. Health Aging.

[25] Nagin D.S. (2014). Group-based trajectory modeling: An overview. Ann. Nutr. Metab..

[26] National Institutes of Health, National Institute on Aging, Intramural Research Program Healthy Aging in Neighborhoods of Diversity across the Life Span. https://handls.nih.gov/02Protocol.htm.

[27] US Department of Health and Human Services The 2004 HHS Poverty Guidelines. https://aspe.hhs.gov/2004-hhs-poverty-guidelines.

[28] Beydoun M.A., Noren Hooten N., Maldonado A.I., Beydoun H.A., Weiss J., Evans M.K., Zonderman A.B. (2022). Body mass index and allostatic load are directly associated with longitudinal increase in plasma neurofilament light among urban middle-aged adults. J. Nutr..

[29] Lafortune L., Martin S., Kelly S., Kuhn I., Remes O., Cowan A., Brayne C. (2016). Behavioural Risk Factors in Mid-Life Associated with Successful Ageing, Disability, Dementia and Frailty in Later Life: A Rapid Systematic Review. PLoS ONE.

[30] Stenholm S., Strandberg T.E., Pitkala K., Sainio P., Heliovaara M., Koskinen S. (2014). Midlife obesity and risk of frailty in old age during a 22-year follow-up in men and women: The Mini-Finland Follow-up Survey. J. Gerontol A Biol. Sci. Med. Sci..

[31] Steinfeldt L., Anand J., Murayi T. (2013). Food reporting patterns in the USDA Automated Multiple-Pass Method. Procedia Food Sci..

[32] U.S. Department of Agriculture Food and Nutrient Database for Dietary Studies. https://www.ars.usda.gov/northeast-area/beltsville-md-bhnrc/beltsville-human-nutrition-research-center/food-surveys-research-group/docs/fndds-download-databases/.

[33] Shivappa N., Steck S.E., Hurley T.G., Hussey J.R., Hebert J.R. (2014). Designing and developing a literature-derived, population-based dietary inflammatory index. Public Health Nutr..

[34] Morley J.E., Malmstrom T.K., Miller D.K. (2012). A simple frailty questionnaire (FRAIL) predicts outcomes in middle aged African Americans. J. Nutr. Health Aging.

[35] Radloff L.S. (1977). The CES-D Scale:A Self-Report Depression Scale for Research in the General Population. Appl. Psychol. Meas..

[36] Jones B.L., Nagin D. (2013). A Stata Plugin for Estimating Group-Based Trajectory Models. Sociol. Methods Res..

[37] Jones B.L., Nagin D.S. (2007). Advances in group-based trajectory modeling and an SAS procedure for estimating them. Sociol. Methods Res..

[38] Shivappa N., Stubbs B., Hebert J.R., Cesari M., Schofield P., Soysal P., Maggi S., Veronese N. (2018). The Relationship Between the Dietary Inflammatory Index and Incident Frailty: A Longitudinal Cohort Study. J. Am. Med. Dir. Assoc..

[39] Millar C.L., Dufour A.B., Shivappa N., Habtemariam D., Murabito J.M., Benjamin E.J., Hebert J.R., Kiel D.P., Hannan M.T., Sahni S. (2022). A proinflammatory diet is associated with increased odds of frailty after 12-year follow-up in a cohort of adults. Am. J. Clin. Nutr..

[40] Resciniti N.V., Lohman M.C., Wirth M.D., Shivappa N., Hebert J.R. (2019). Dietary Inflammatory Index, Pre-Frailty and Frailty among Older US Adults: Evidence from the National Health and Nutrition Examination Survey, 2007–2014. J. Nutr. Health Aging.

[41] Laclaustra M., Rodriguez-Artalejo F., Guallar-Castillon P., Banegas J.R., Graciani A., Garcia-Esquinas E., Lopez-Garcia E. (2020). The inflammatory potential of diet is related to incident frailty and slow walking in older adults. Clin. Nutr..

[42] Satia J.A. (2009). Diet-related disparities: Understanding the problem and accelerating solutions. J. Am. Diet Assoc..

[43] Tanaka T., Talegawkar S.A., Jin Y., Bandinelli S., Ferrucci L. (2021). Association of Adherence to the Mediterranean-Style Diet with Lower Frailty Index in Older Adults. Nutrients.

[44] Shivappa N., Steck S.E., Hurley T.G., Hussey J.R., Ma Y., Ockene I.S., Tabung F., Hebert J.R. (2014). A population-based dietary inflammatory index predicts levels of C-reactive protein in the Seasonal Variation of Blood Cholesterol Study (SEASONS). Public Health Nutr..

[45] US Food and Drug Administration Final Determination Regarding Partially Hydrogenated Oils (Removing Trans Fat). https://www.fda.gov/food/food-additives-petitions/final-determination-regarding-partially-hydrogenated-oils-removing-trans-fat.

