# Supplementary material for: Pro-Inflammatory Diets Are Associated with Frailty in an Urban Middle-Aged African American and White Cohort
Source: Nutrients. 2023 Oct 29;15(21):4598. doi: 10.3390/nu15214598 (PMC10648548; doi:10.3390/nu15214598)
Supplement: Supplementary file 1 [file nutrients-15-04598-s001.zip › nutrients-2689097-supplementary.pdf]

Supplementary Tables

Table S1. Group-based Trajectory Modeling Output for Frailty

Group	Parameter	Estimate	Standard Error	T for HO: Parameter=0	Prob > |T|
1	Intercept	-26.03033	31.27852	-0.832	0.4053
	Linear	0.71914	1.06835	0.673	0.5009
	Quadratic	-0.00548	0.00909	-0.603	0.5464
2	Intercept	-1.96860	0.45328	-4.343	0.0000
	Linear	0.04938	0.01730	2.855	0.0043
	Quadratic	-0.00032	0.00016	-1.943	0.0520
Group membership					
1	%	20.16246	2.11555	9.531	0.0000
2	%	79.83754	2.11555	37.738	0.0000

BIC= -6464.62 (N=6615) BIC= -6461.73 (N=2901) AIC = -64440.83 II=-6433.83

Parameter estimates for adding risk factors
-26.03033, 0.71914, -0.00548, -1.96860, 0.04938, -0.00032, 1.37617

Parameter estimates
-26.03033, 0.071914, -0.00548, -1.96860, 0.04938, -0.00032,20.16246,79.83754

Entropy = 0.593

Table S2. Group-based Trajectory Modeling Output for Dietary Inflammatory Index

Group	Parameter	Estimate	Standard Error	T for HO: Parameter=0	Prob > ǀTǀ
1	Intercept	0.17337	0.54738	0.317	0.7515
	Linear	-0.00803	0.01029	-0.780	0.4352
2	Intercept	5.17445	0.22346	23.156	0.0000
	Linear	-0.01175	0.00393	-2.988	0.0028
3	Intercept	3.90312	0.20879	18.694	0.0000
	Linear	-0.02447	0.00390	-6.266	0.0000
1	Sigma	1.82001	0.07205	25.261	0.0000
2	Sigma	1.25981	0.04206	29.955	0.0000
3	Sigma	1.69270	0.02735	61.884	0.0000
Group membership					
1	%	10.10761	1.42464	7.095	0.0000
2	%	32.27672	3.33842	9.668	0.0000
3	%	57.61567	2.85355	20.191	0.0000

BIC= 13247.12 (n=6365), BIC=13242.80 (N=2901), AIC = 13209.95 II = -13198.95

Parameter estimates for adding risk factors
0.17337, -0.00803, 5.17445, -0.01175, 3.90312, -0.02447, 1.82001, 1.25981, 1.69270, 1.16106, 1.74051

Parameter estimates
0.17337, -0.00803, 5.17445, -0.01175, 3.90312, -0.02447, 1.82001, 1.25981, 1.69270, 10.10761, 32.27672, 57.61567

Entropy = 0.594

Table S3. Percentage of sample by group trajectory and mean (±SE) DII scores by group trajectory for each HANDLS study visit (n=2901)

Diet Quality		DII Scores by Trajectory		
	% sample	Visit 1	Visit 2	Visit 3
		X±SE	X±SE	X±SE
Low quality (High DII)	32.3	5.07±0.04	4.70±0.04	4.48±0.05
Medium quality (Medium DII)	57.6	2.85±0.05	2.49±0.04	2.11±0.04
High quality (Low DII)	10.1	-0.69±0.13	-0.55±0.12	-0.88±0.11

Notes: DII Dietary inflammatory index; HANDLS, Healthy Aging in Neighborhoods of Diversity Across the Life Span; SE, Standard Error

Table S4. Association of DII GBTM trajectories with frailty GBTM trajectories, by sex. Multiple Logistic Regression models, HANDLS study (N=1,349, N′=2,065, n=549)

Regression Models	Males N=1,261			Females N=1,640		
	Coefficient	SE	p	Coefficient	SE	p
**Model 1**						
Pre-frail or frail	Base outcome			*Base outcome*		
Remaining non-frail trajectory=main outcome						
DII trajectory	Base Group 1					
Medium vs. High DII	0.673	0.184	<0.001	0.662	0.150	<0.001
Low vs. High DII	1.734	0.251	<0.001	1.391	0.241	<0.001
Age *v*1	0.010	0.008	0.210	0.009	0.007	0.178
Race	0.360	0.145	0.013	0.209	0.135	0.122
Poverty status	-0.803	0.157	<0.001	-0.959	0.145	<0.001
**Model 2**						
Pre-frail or frail	Base outcome			*Base outcome*		
Remaining non-frail trajectory=main outcome						
DII trajectory	Base Group 1			Base Group 1		
Medium vs. High DII	0.589	0.189	0.002	0.574	0.156	<0.001
Low vs. High DII	1.518	0.262	<0.001	1.018	0.261	<0.001
Age *v*1	0.009	0.008	0.297	0.012	0.007	0.093
Race	0.406	0.153	0.008	0.237	0.142	0.095
Poverty status	-0.678	0.164	<0.001	-0.786	0.150	<0.001
Education	Base < High School			Base < High School		
High School	-0.096	0.319	0.764	0.782	0.435	0.072
> High School	0.002	0.331	0.995	0.986	0.445	0.027
Current smoker *v*1	-0.579	0.178	0.002	-0.540	0.174	0.059
Drug User *v*1	-0.372	0.198	0.061	-0.529	0.276	0.003
Allostatic load	-0.184	0.072	0.016	-0.228	0.060	<0.001

Notes: N=Number of subjects included in the analysis; N′=Number of observations included in the analysis, n=total number of incident frail/pre-frail. Coefficients are the Loge(HR), SE is the standard error of the coefficient. CI, Confidence Interval; DII, Dietary inflammatory index; HANDLS, Healthy Aging in Neighborhoods of Diversity Across the Life Span; HR, Hazard Ratio; LCL, Lower Confidence Limit of the 95% CI of HR; UCL, Upper Confidence Limit of the 95% CI of HR; *v*1, *visit* 1

Table S5. Multiple Logistic Regressions of the association of diet quality and frailty by race

Regression Models	African American adults			White adults		
**Model 1**	Coefficient	SE	p	Coefficient	SE	p
Pre-frail or frail	Base outcome			*Base outcome*		
Remaining non-frail trajectory=main outcome						
DII trajectory	Base Group 1			Base Group 1		
Medium vs. High DII	0.581	0.141	<0.001	0.834	0.207	<0.001
Low vs. High DII	1.190	0.253	<0.001	1.949	0.254	<0.001
Age *v*1	0.004	0.007	0.525	0.163	0.008	0.047
Sex, Male	0.1956	0.122	0.110	0.029	0.152	0.850
Poverty status	-0.933	0.128	<0.001	-0.755	0.189	<0.001
**Model 2**	Coefficient	SE	p	Coefficient	SE	p
Pre-frail or frail	Base outcome			*Base outcome*		
Remaining non-frail trajectory=main outcome						
DII trajectory	Base Group 1			Base Group 1		
Medium vs. High DII	0.522	0.146	<0.001	0.696	0.214	0.001
Low vs. High DII	0.990	0.265	<0.001	1.622	0.273	<0.001
Age *v*1	0.006	0.007	0.446	0.018	0.009	0.037
Sex, Male	0.345	0.131	0.008	0.158	0.158	0.318
Poverty status	-0.800	0.134	<0.001	-0.592	0.196	0.003
Education	Base < High School			Base < High School		
High School	0.539	0.373	0.149	0.014	0.342	0.904
> High School	0.686	0.381	0.072	0.176	0.360	0.625
Current smoker *v*1	-0.634	0.146	<0.001	-0.442	0.206	0.038
Drug User *v*1	-0.471	0.193	0.016	-0.332	0.276	0.231
Allostatic load	-0.207	0.067	0.005	-0.210	0.072	0.005

Notes: DII, Dietary inflammatory index; *v*1, *visit* 1.

Table S6. Mixed-effect regression of the association of *v*1 frailty status and DII continuous score, HANDLS study

DII, N=2,675, k=2.2 observations/participant	Coefficient	SE	p
**Model 1**			
Time	-0.101	0.012	<0.001
v1 Frailty			
Pre-frail	-0.413	0.088	<0.001
Frail	0.972	0.144	<0.001
**Time *x* Pre-frail**	**0.015**	**0.013**	**0.229**
**Time *x* Frail**	**-0.029**	**0.022**	**0.182**
Age *v*1	-0.004	0.004	0.265
Time *x* Age	0.002	0.001	0.013
Sex, Male	-0.500	0.082	<0.001
Time *x* Sex	-0.0004	0.0112	0.971
Race, African American	0.536	0.082	<0.001
Time *x* Race	-0.012	0.012	0.335
Poverty status	0.136	0.084	0.107
Time *x* Poverty status	0.045	0.012	<0.001
**Model 2**			
Time	-0.070	0.027	0.010
v1 Frailty			
Pre-frail	0.280	0.086	0.001
Frail	0.760	0.142	<0.001
**Time *x* Pre-frail**	**0.012**	**0.013**	**0.332**
**Time x Frail**	**-0.031**	**0.022**	**0.165**
Age *v*1	-0.006	0.004	0.207
Time *x* Age	0.0015	0.0006	0.024
Sex, Male	-0.585	0.080	<0.001
Time *x* Sex	0.0002	0.0118	0.989
Race, African American	0.549	0.081	<0.001
Time *x* Race	-0.011	0.012	0.361
Poverty status	-0.077	0.084	0.364
Time *x* Poverty status	0.046	0.123	<0.001
v 1 education- base			
High School	-0.342	0.163	0.036
> High School	-1.150	0.171	<0.001
Time *x* high school	-0.044	0.025	0.073
Time *x* > high school	-0.021	0.026	0.415
Current smoker *v*1	0.411	0.087	<0.001
Time *x* smoker	0.003	0.013	0.811
Drug User *v*1	-0.207	0.110	0.061
Time *x* drug user	0.009	0.016	0.593
Allostatic load	0.058	0.032	0.068
Time *x* allostatic load	0.006	0.006	0.279

Notes: DII, Dietary inflammatory index; HANDLS, Healthy Aging in Neighborhoods of Diversity Across the Life Span; *v*1,*visit* 1.
